# Factors Influencing Host Plant Choice and Larval Performance in *Bactericera cockerelli*

**DOI:** 10.1371/journal.pone.0094047

**Published:** 2014-04-07

**Authors:** Sean M. Prager, Isaac Esquivel, John T. Trumble

**Affiliations:** Department of Entomology, University of California Riverside, Riverside, California, United States of America; Federal University of Viçosa, Brazil

## Abstract

Among the many topics of interest to ecologists studying associations between phytophagous insects and their host plants are the influence of natal host plant on future oviposition decisions and the mechanisms of generalist versus specialist host selection behavior. In this study, we examined the oviposition preferences, behavior and larval development of the tomato/potato psyllid, *Bactericera cockerelli*. By rearing psyllids with two distinct geographically-linked haplotypes on different host plants, we were able to examine the role of natal host plant and potential local adaptation on host plant usage. Choice bioassays among three host species demonstrated that psyllids from California had clear preferences that were influenced by natal plant. We further found that patterns in choice bioassays corresponded to observed feeding and movement responses. No-choice bioassays demonstrated that there is little to no association between development and host-plant choice for oviposition, while also indicating that host choice varies between haplotypes. These findings support the concept that mothers do not always choose oviposition sites optimally and also add support for the controversial Hopkins' host selection principle.

## Introduction

Studies suggest a general pattern in which most herbivores have a diet restricted to one or a few host plant species [1], [2], the presumed result of coevolution between these species [3]–[5]. However, some species are generalists that use multiple plants for feeding and/or oviposition [1]. This plasticity is important for many herbivorous insects, and may be an evolutionary adaptation permitting them to adapt to variable environments [6]–[10].

Unfortunately, the mechanisms of host-plant choice are not always easily delineated. Many aspects have been considered as determinants or factors in an herbivore's host range including ability to detect hosts, larval physiology, natural enemies, and reproductive behaviors [2], [11]–[13]. Although there are theories to explain host plant choice in herbivores, probably the most common explanation is the ‘mother knows best’ principle [14], [15], which is alternatively known as the optimal oviposition theory [16], [17] and classically as the preference–performance hypothesis [18]. These hypotheses are based on the concept that juvenile life stages are frequently limited in their ability to move among plants, and therefore a female should choose the best possible host plant on which to oviposit and for her offspring to develop. This concept is therefore evaluated in terms of juvenile performance, whose correlates include survival rates, development to a particular stage or in some instances morphological size, but is frequently evaluated as developmental time [19]. However, it is important to consider that a female may make host choice decisions to maximize her own performance rather than that of her offspring [19]. Such decisions may be based on factors influencing her survival, such as nutritional quality of the plant [20]. Additionally, factors such as risk spreading, predation, and proximity to other resources may all influence oviposition choices [17], [19], [21], [22]. Because of these associations, studies typically examine either ‘preference traits’ that determine willingness to use a host plant, or ‘performance traits’ that encompass the ability to grow, survive, and develop on a host plant [23]. In these alternative scenarios, insect species fail to make the seemingly optimal choice to oviposit on the plant species that will result in optimal growth and development for their offspring [14].

Hopkins' host selection principle is the rather controversial observation that “a species which breeds on two or more hosts will prefer to continue to breed on the host to which it has become adapted” [24]. The concept was first applied to mountain pine beetles (*Dendroctonus monticolae* Coleoptera: Scolytidae Hopkins), but has since been refined many times [25]–[28]. Further, there are numerous empirical studies both supporting [29] and refuting [30] the validity of the concept. Most studies have demonstrated that experience as an imago following emergence from the pupal stage is the key factor in developing this type of preference. This has been termed the neo-Hopkins' principle [25]. Notably, this concept would be restricted to holometabolous insects in which the brain is likely to be restructured during metamorphosis.

Psylloidea (jumping plant lice) typically have narrow host ranges consisting of a single genus or family [31]–[33]. This is not the case with the potato psyllid, *Bactericera cockerelli* Šulc, which has a reported host range of over 40 species in 20 families and a general preference for the Solanaceae [34]–[36]. Wallis [35] reports that potato psyllids can ‘breed’ on plants from three families, *Solanaceae*, *Convolvulaceae* and *Lamiaceae*. This includes the important vegetable crops: bell pepper ( = capsicum) (*Capsicum annum* L.), potato (*Solanum tuberosum* L.), eggplant (*Solanum melongena* L.), and tomato (*Solanum lycopersicum* L.). However, some discrepancies do exist. Knowlton and Thomas [37] reported failure of potato psyllid nymphs to mature when presented with bell pepper, while Yang and Liu [38] report relative success on bell pepper, as do Liu and Trumble [39]. Additionally, Liu et al. [40] reported that geography influences life history of potato psyllids collected in California versus those collected in Texas, but only with tests on tomato. It has subsequently been demonstrated that these two geographic locations (California and Texas) contain genetically different potato psyllid haplotypes [41]–[43]. Differential patterns of host use are not exclusive to bell pepper, as it has been reported that host plant (eggplant or bell pepper) influences fecundity of Texas collected potato psyllids, with more eggs laid on bell pepper [44]. Additionally, both tomato cultivar [39] and potato variety (Prager et al., in press) influence the attractiveness to potato psyllids. Lastly, Prager et al. (in press) demonstrated that the developmental stage of potato plants influences attractiveness for feeding. It has been suggested that the physiological state of the plants is responsible for this variation in attractiveness.

*Bactericera cockerelli* is a pest for three distinct reasons. First, honeydew accumulation in some crops, especially pepper, results in sooty mold that interferes with photosynthesis and can contaminate the fruit. Second, *B. cockerelli* feeding on tomato and potato can result in an infection of unknown cause called ‘psyllid yellows’ [36]. Third, *B. cockerelli* transmits *Candidatus* Liberibacter solanacearum (syn. “Ca. L. psyllaurous”) (CLso) [45]–[47] a bacterial pathogen that infects and causes disease in multiple solanaceous plants including tomatoes, potatoes, peppers, eggplants and tobacco [45], [48]–[50].

In this study, we examined the host plant preference and performance of *Bactericera cockerelli* on three plant species known to serve as hosts: the closely related potato and tomato, and the more distantly related bell pepper. We used choice bioassays to determine oviposition and settling preferences among plant species. We then follow up these assays with observations of feeding, cleaning (grooming of the wings and head with the legs) and movement and no-choice bioassays to examine performance on preferred and non-preferred hosts. Additionally, we examined psyllids reared on multiple host plants to examine the effect of natal plant on host-choice behavior and performance. Combined, these experiments are the first to directly examine host plant preferences for a psyllid with a wide host range, and among the first to relate these preferences to performance on host plants. Additionally, this is one of the first studies to examine the effect of haplotype on psyllid host plant preferences.

## Materials and Methods

### Insects and plants

Studies were conducted using three plant species: tomato (*Solanum lycopersicum esculentum* L., variety ‘Yellow Pear’), bell pepper (*Capsicum annuum* L., variety ‘Cal Wonder’), and potato (*Solanum tuberosum* L., variety ‘Atlantic’), commonly used for laboratory studies and rearing of potato psyllids. Plants for colony maintenance and bioassays were all maintained under identical conditions. Tomato and bell pepper plants were grown from seed in 10.16 cm pots with UC soil mix [51], fertilized with Miracle Gro nutrient solution (Scotts Company, Marysville, OH) at label rate, and watered daily. Potatoes were grown from seed pieces in 15 cm diameter pots with UC soil mix, watered and fertilized with Miracle Gro nutrient solution *ad libitum.*

Original insect source material for these experiments came from two colonies each maintained at the University of California, Riverside for a minimum of five years. The first colony (henceforth ‘Texas’) was collected from tomato and potato fields near Weslaco, TX and was maintained on tomato. The second colony (‘California’) was collected from fields of bell pepper in Orange County, California and was maintained on bell pepper. Both colonies were tested for genetic haplotype using the methods of Swisher et al. [43]. California was confirmed to be the ‘western’ haplotype, while Texas was the ‘central’ haplotype. Both colonies were maintained in multiple mesh tents (Bugdorm, BioQup, Rancho Dominguez, CA) at conditions of 21–26°C and 40–60% relative humidity, and maintained under ambient light conditions. Texas and California populations were housed in spatially distinct locations to prevent cross-contamination. In order to conduct no-choice bioassays that considered the effect of “natal” host plant, colonies of *B. cockerelli* were established by transferring nymphs and adults from the main source colonies (California or Texas) into cages with the alternate host plants (potato, tomato, or bell pepper). We were unable to establish a colony from California reared on potato, thus that combination is excluded from all studies. To ensure that all psyllids used in bioassays were from eggs laid on the new host plant, colonies were maintained for a minimum of three full generations prior to use. All psyllids used in these studies were from colonies infected with CLso, and the presence of CLso was confirmed periodically via a Taqman based real time PCR assay with the methods of Butler et al. [52]. Voucher specimen from the *B. cockerelli* colonies have been deposited in the University of California, Riverside Entomology Research Museum.

### Three-choice bioassays

Three choice bioassays were conducted in arenas consisting of two pieces of 5 mm thick foam each glued to one side of a square 25×25 cm piece of plastic. Each piece of foam, had a 20 cm hole cut into the center. Foam pieces were glued to each other on the corners and along all sides, with the exception of a 5 cm long segment through which plant stems were placed. The resulting arena consisted of a sandwich with the two foam pieces on the inside and the clear plastic on the outside. In setting up bioassays, one stem with one terminal leaf or leaflet each from a whole intact pepper, potato, and tomato plant was haphazardly assigned to a side of the arena; the fourth side contained no plants and was taped shut. Leaves were placed into the arena through the 5 cm slit, which were then sealed using metal ‘duckbill’ hair clips (Supporting Information S1).

To perform bioassays, psyllids were removed from colonies, separated into size 1 gelatin pill capsules (CapsluCN International Co., China) and sorted by sex. Five male: female pairs of post-teneral psyllids were then aspirated into the arenas. In choice bioassays, *B. cockerelli* typically take 48 h to acclimate and settle onto plants (Prager, unpublished data). Consequently, the location of all potato psyllids (on pepper, tomato or potato) was recorded 48, 72 and 96 h after being placed into the arena. Following the 96 h psyllid count, potato psyllids were removed and the numbers of eggs on each leaf were counted. Three choice bioassays were replicated 10 times each with *B. cockerelli* from California maintained on tomato and on pepper.

In these experiments, oviposition preference was examined using generalized linear models (GLM) with negative binomial probability distributions. The dependent variable was number of eggs on each plant with fixed terms for natal and host plant species.

Since a psyllid can only be on a single plant at a time, location data are somewhat correlated. Consequently, settling behavior (the plants on which psyllids were observed) was first examined using “permutational MANOVA” (PERMANOVA), a method of partitioning sums of squares analogous to parametric MANOVA [53], [54] but robust to non-normality. The model examined included the number of psyllids on a given host plant summed across days as a response variable and an independent factor of natal host plant. This was then followed up using a generalized linear model with a negative binomial probability distribution. The response variable was again summed psyllids and the predictor was the exposure plant.

### Behavioral observations

Behavioral observations were conducted according to the methods of Liu and Trumble [55]. Briefly, assays were conducted on whole intact plants in arenas created by layering a 9 by 12 cm Plexiglass rectangle stage, a 1 by 3 by 6 cm square piece of foam with a 2 cm hole and a clear glass cover. This resulted in a chamber that contained the leaflet and psyllid while providing visual access to the leaflet and the psyllid. A newly emerged post-teneral adult female was introduced to the arena and allowed a 5 min acclimation period to adjust to the microenvironment. Following the acclimation period, a 15 min observation period began during which we recorded behaviors routinely used in studies assessing insect activity [56]. Studies by Liu and Trumble [55] indicated that the 15-min observation period was adequate for the psyllids to exhibit the complete range of behaviors. Because the time period is adequate for multiple occurrences of all behaviors, the insects will have sufficient time to abandon the leaf if it is not acceptable. The observations were recorded using the Observer XT (Noldus Information Technologies, Wageningen, Netherlands) software program, which records data on the cumulative duration of each behavior as well as the number of occurrences of each behavior. Specific behaviors recorded included cleaning, jumping, resting, off leaf (exiting or abandoning the leaf surface), walking, probing and feeding. Feeding in *B. cockerelli* requires accessing the phloem, which can take in excess of 4 hours from insertion of the proboscis [57]. Consequently, we measure behaviors (feeding and probing) that are part of the series of behaviors associated with feeding rather than entire feeding bouts. Each natal plant by test plant combination was replicated 10 times using only psyllids collected in California.

Since all behaviors were recorded simultaneously within an observation period, and since some are mutually exclusive, it is not possible to assume independence among behavioral responses. Additionally, many of the behaviors examined as durations of 15-minute observation periods had non-normal distributions. Consequently, we analyzed these data using PERMANOVA. The specific model featured fixed effects for the exposure plant and the natal plant in addition to an interaction term. We were also unable to transform frequencies to normality and again used PERMANOVA to look for an overall effect. Significant effects on frequencies of behaviors were followed up by generating individual univariate GLMs fit with either a negative binomial or Poisson probability distribution, chosen based on Akaike information criteria (AIC) values and evaluated with an adjusted p-value calculated with Bonferroni's method.

### Performance/development bioassays

To examine patterns of performance and development, we conducted no-choice bioassays by caging two male: female pairs of post-teneral *B. cockerelli* onto a terminal leaflet of a plant using white 10.16×15.25 cm mesh sachet party favor bags (JoAnn Fabric and Craft Stores). One sachet and pair of *B. cockerelli* was used per plant. Cages were placed on a leaf on the top third of plants. Plants were maintained in a climate controlled insect rearing room at 21–26°C and 40–60% relative humidity for 48 hours after which *B. cockerelli* were removed and the number of eggs was counted. Plants were then inspected daily for the numbers of eggs, small (1^st^ or 2^nd^ instar) nymphs, large (3^th^, to 5^th^ instar) nymphs, and adults until all potato psyllids either removed as adults or died. Instar was determined by approximating body width or presence of wing pads as in Liu and Trumble [39]. No-choice bioassays were replicated a minimum of 10 times with each combination of natal plant (pepper, potato, tomato) by test plant (pepper, potato, tomato) by haplotype of *B. cockerelli* colony (California or Texas). The sole exception is that no colony of the California collected *B. cockerelli* could be established on potato and so the relevant combinations were not tested.

Patterns of oviposition were tested using GLM with a negative binomial distribution. The model included the numbers of eggs as a response variable and the independent factors: natal host plant, exposure host plant, and haplotype, in addition to all interactions. Models were subsequently simplified via backwards selection.

We examined the proportion of eggs that hatched using a series of GLMs. Analyses were conducted on the arcsine square-root of the proportion of eggs that hatched, as the proportion that hatched was non-normally distributed. The transformed hatch proportion was analyzed using a model that included terms for: natal host plant, exposure host plant, and haplotype, in addition to all interactions. Models were subsequently simplified via backwards selection.

To determine the effect of host plant on psyllid development, we calculated growth index (GI), defined as the sum of the highest growth stage individuals would achieve in an ideal control population, using the method of Zhang et al. [58]. GI ranges from zero to one, with one indicating most individuals survived to adult while zero indicated no insects survived beyond the first stage. This model was fit using general additive modeling (GAM) [59] because GI was slightly bimodal. Since GAM cannot account for interactions between terms, we performed a follow-up analysis using GLM. Initially, we examined a model with the terms natal host plant, exposure host plant, and haplotype. This model was subsequently simplified using reverse model selection. To cope with non-normality of GI, we analyzed both raw GI and GI replaced with ranks. Since model results were nearly identical, we report results from the untransformed analysis. Finally, it may be expected that GI would be correlated with numbers of eggs, since females should lay more eggs when her offspring will perform better. We examined this using a generalized linear model with a negative binomial probability distribution. We initially used a full model with the dependent variable eggs and the factors GI, exposure plant, natal plant, haplotype, and all interactions. This was simplified using stepwise backward selection and eventually resulted in a model that included all the main effect terms and the interaction of exposure plant and haplotype. It is not possible to calculate a standard R^2^ in for this type of GLM model, and so the adjusted pseudo-R^2^ was used as a measure of variation explained.

### Statistical Analyses

All analyses were performed using the R statistical language version 2.3.0 (R Development Core 2008). Linear mixed-effects models with negative binomial error were implemented using MASS package [60]. Type II analysis of variance tables were calculated using the car package [61]. Permutated MANOVA was implemented using the adonis function of the vegan package [53], [62], and 1000 permutations. GAM was implemented with the R package mgcv [59]. Adjusted pseudo-R^2^ values were calculated using the R package vegan [62], [63].

## Results

### Three-choice bioassays

**Figure 1:**
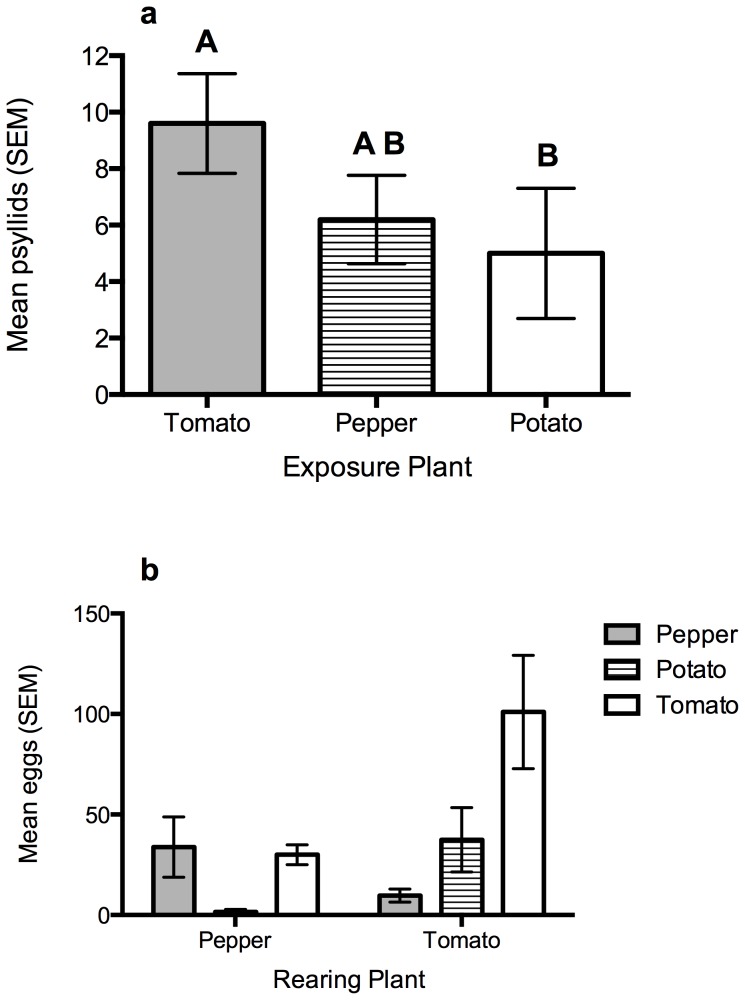
Oviposition and settling behavior in three-choice bioassays. **a**. The mean number (standard error) of psyllids on pepper, potato and tomato in three-choice bioassays. Identical capital letters above bars indicate no significant difference in contrasts. **b**. The mean number of eggs on pepper, potato and tomato in three-choice bioassays, when reared on either pepper or tomato.

Analyses indicated no effect of natal plant on *B. cockerelli* settling behaviors (PERMANOVA: F_1, 18_ = 0.7, *P* = 0.5). However, follow up analyses demonstrated an overall preference for settling among the different plant species in the choice arena (χ^2^_2_ = 8.9, *P*<0.05) (Figure 1a). Examination of contrasts indicates that more *B. cockerelli* settle onto tomatoes than onto potatoes (Z = −2.8, *P*<0.001), but that they are equally likely to settle on pepper as tomato (Z = −1.9, *P*<0.054) and equally likely to settle onto potato as pepper (Z = −0.96, *P*<0.34).

In examining host-plant choice for oviposition, we found a significant effect of the natal host plant (χ^2^_1_ = 6.3, *P*<0.05) and a significant natal by test plant interaction (χ^2^_2_ = 8.4, *P*<0.02), while the effect of the exposure plant species was slightly insignificant (χ^2^_2_ = 5.4, *P*<0.067) (Figure 1b). Similar to the patterns displayed in settling behavior, *B. cockerelli* reared on pepper oviposited less on potato than the other plants. Conversely, those reared on tomatoes laid more eggs on tomato and laid fewer eggs on pepper.

### Behavioral observations

**Figure 2:**
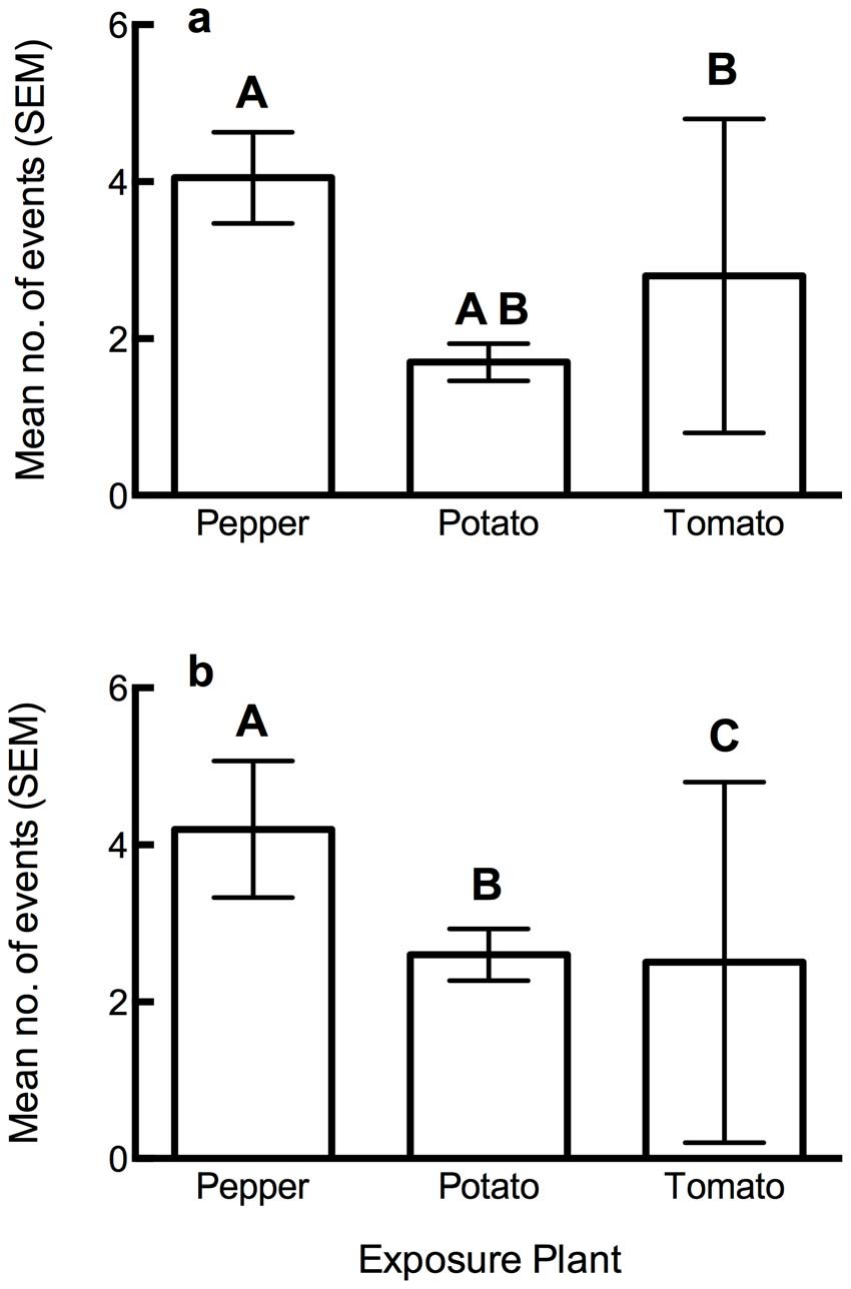
Behavioral responses to different host plants. Mean number of resting (**a**) or walking events (**b**) observed when *B. cockerelli* are exposed to pepper, potato or tomato. Identical capital letters above bars indicate no significant difference in contrasts.

When behavioral events were examined as the duration of a 15 min. observation period during which they were performed, there was no effect of exposure plant (PERMANOVA: F_2, 77_ = 1.65, *P* = 0.155) or of the natal host plant (F_1, 77_ = 0.63, *P* = 0.5) on behavior; however, the interaction term was significant (F_1, 77_ = 4.9, *P*<0.05). When the numbers of behaviors performed (frequencies) were tested, there was a significant main effect of the exposure plant (PERMANOVA: F_2, 77_ = 3.6, *P*<0.001) and also a significant interaction between the host and natal plants (F_1, 77_ = 4.4, *P*<0.001); there was no significant main effect of the natal host plant (F_1, 77_ = 1.08, *P* = 0.36). When these significant effects were followed up using individual tests, there was a significant effect of the test plant on resting (χ^2^_2_ = 16.4, *P*<0.0001) (Figure 2a) and walking (χ^2^_2_ = 7.9, p<0.05) (Figure 2b). When examined using individual contrasts, differences in walking were due to differences with pepper while differences in resting were between: pepper and potato, pepper and tomato, and potato and tomato. *B. cockerelli* exposed to potato rested and walked less than those exposed to tomato or pepper. Those exposed to pepper walked more than those exposed to other plant species. There was also a significant interaction of natal and exposure plants on probing (χ^2^_2_ = 4.2, *P*<0.05), feeding (χ^2^_2_ = 5.7, *P*<0.05) and jumping (χ^2^_2_ = 6.0, *P*<0.05). Overall, these patterns would suggest that psyllids are more apt to settle and search for feeding sites on the plant species they were reared on.

### No-choice bioassays

**Figure 3:**
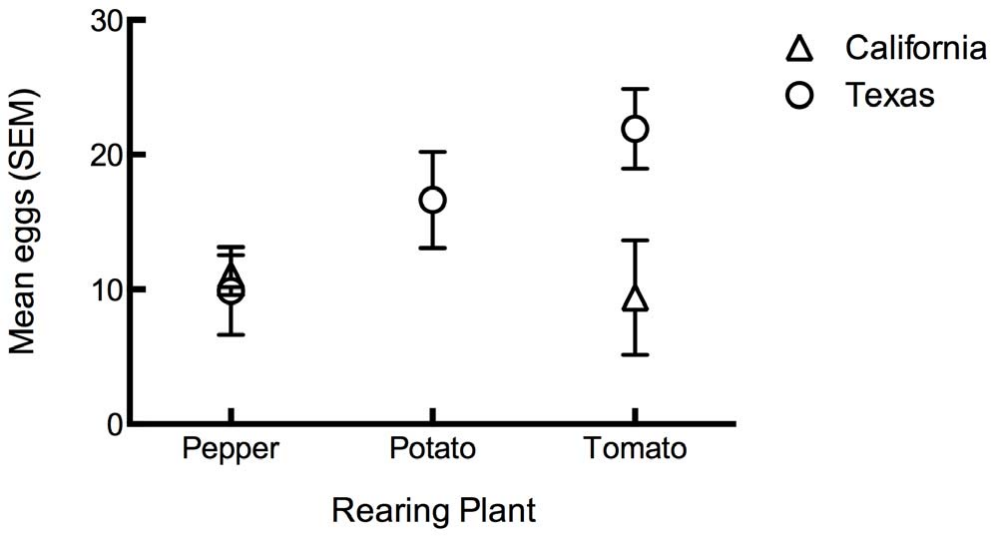
Mean eggs laid on pepper, potato, and tomato by *B. cockerelli* reared on pepper, tomato, and potato when collected in California or Texas.

*Bactericera cockerelli* plant preference for oviposition was examined with a model that included individual terms for the natal plant (χ^2^ = 3.4 df = 2, *P* = 0.18), exposure plant (χ^2^ = 2.4 df = 2, *P* = 0.29) and *B. cockerelli* haplotype (χ^2^ = 0.2 df = 1, *P* = 0.6). In the model none of the main effects were significant. However, there was a significant natal plant by haplotype interaction (χ^2^ = 5.3, df = 1, *P*<0.05) (Figure 3). This interaction effect is apparently driven by those instances where *B. cockerelli* were “switched” from the plant on which they were initially collected onto another natal host plant. *B. cockerelli* from Texas reared on tomato laid more eggs than any other combination, while regardless of haplotype, the fewest eggs were laid by psyllids reared on pepper. These results again indicate that haplotype influences oviposition, and that they are particularly sensitive to differences involving bell pepper.

**Figure 4:**
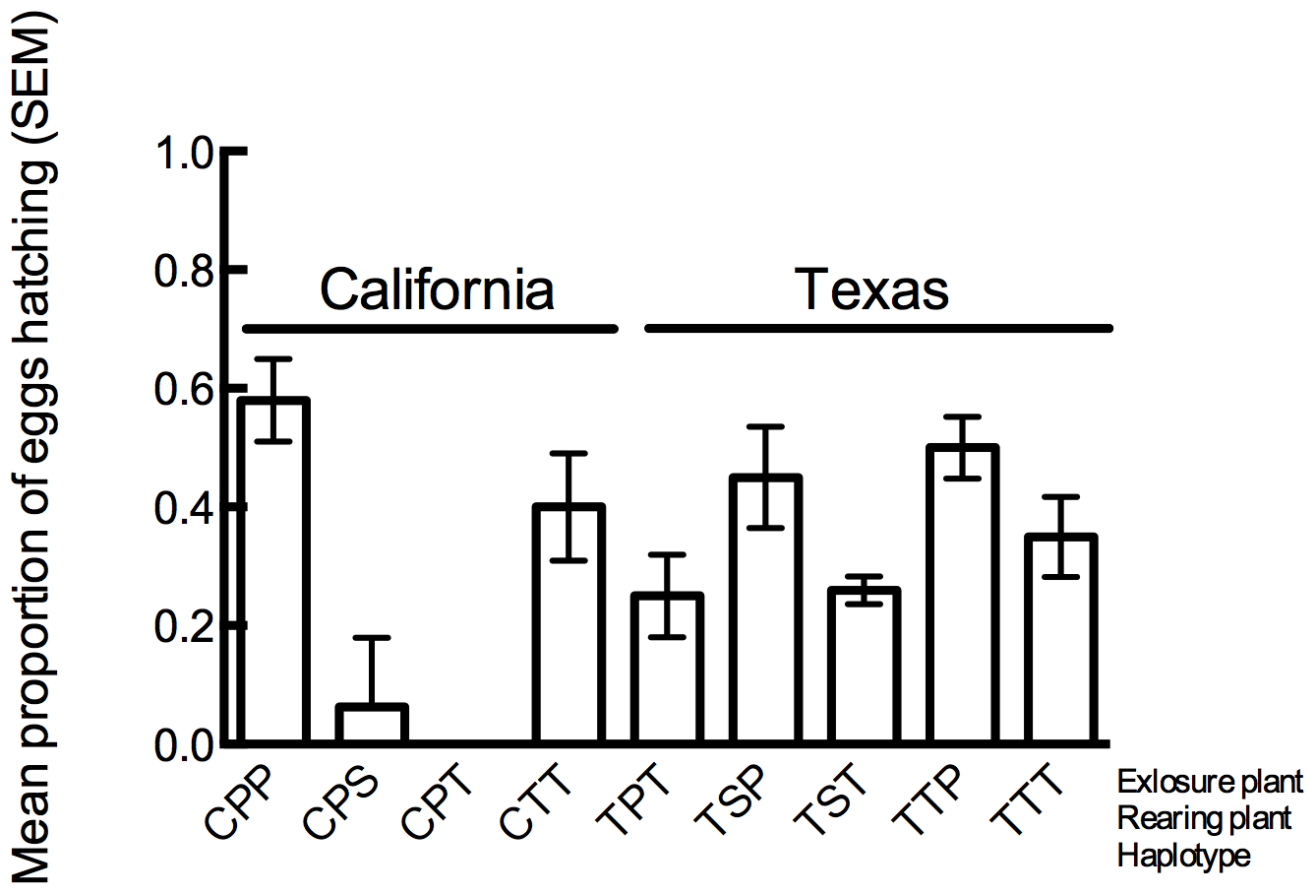
Mean proportion of eggs that hatched. Treatment combinations are abbreviated as: California or Texas (C or T), then natal host plant (T = Tomato, P = Pepper, S = Potato), and then exposure host plant (T = Tomato, P = Pepper, S = Potato).

We found a complicated pattern with respect to the hatching of eggs with multiple significant main effects and interactions (Figure 4). Specifically, there were significant effects of haplotype (χ^2^ = 7.5, df = 1, *P*<0.01) and exposure host plant (χ^2^ = 12.4, df = 2, *P*<0.01). Additionally, there were significant two-way interactions between haplotype and natal plant (χ^2^ = 9.1, df = 1, *P*<0.01) and haplotype and exposure plant (χ^2^ = 9.4, df = 2, *P*<0.01). Among the patterns revealed, eggs laid by *B. cockerelli* from California reared on and exposed to pepper hatched more often than the other California combinations. Also, only California *B. cockerelli* had a combination in which no eggs hatched. These results indicate that a *B. cockerelli* 's natal host influences how successfully her eggs will hatch, but that this effect is also influenced by her haplotype. This may reflect differences in egg quality that may, in turn, reflect differences in nutritional quality among host plant species.

**Figure 5:**
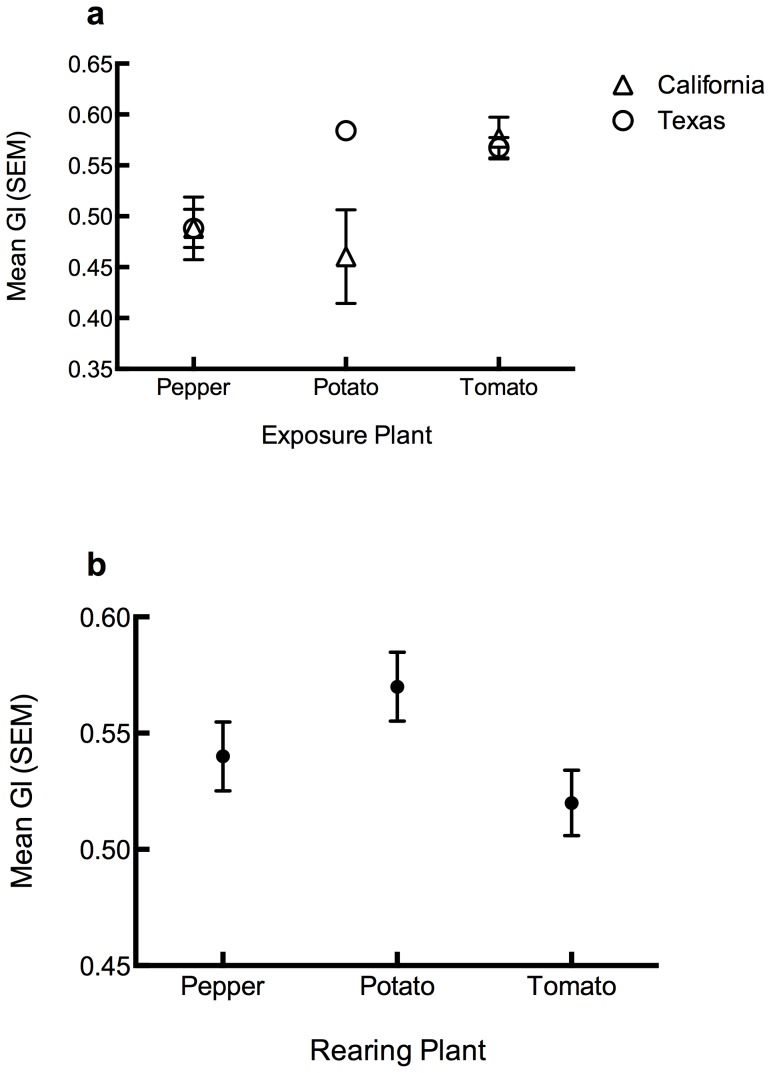
Development of *B. cockerelli.* **a**. Mean growth index for *B. cockerelli* exposed to pepper, tomato, and potato when collected in California or Texas. **b**. Mean GI for *B. cockerelli* reared on pepper, tomato or potato.

To evaluate the performance of *B. cockerelli* on different host plants, we calculated growth index (GI) for each no-choice bioassay combination. This was again examined via GLM and the analyses revealed a significant effect of exposure plant (F = 20.001, df = 2, *P*<0.0001) (Figure 5a), natal plant (F_2_ = 13.221, *P*<0.0001) (Figure 5b), and also haplotype and exposure plant interaction (F_2_ = 12.0, *P*<0.001) (Figure 5b). *B. cockerelli* reared on potato most successfully became adults, while those on tomato were least successful. However, *B. cockerelli* exposed to tomato achieved adulthood most often regardless of haplotype. *B. cockerelli* from California exhibited lower GI values on potato than those from Texas. The fixed main effect of haplotype was not significant (F_1_ = 2.2, *P* = 0.13).

Finally, the numbers of eggs deposited can be significantly explained by growth index. Specifically, there was a significant effect of GI (χ^2^_1_ = 6.7, *P*<0.001), as well as haplotype (χ^2^ = 15.8 df = 1, p<0.0001) and the interaction of exposure plant and haplotype (χ^2^_2_ = 24.5, *P*<0.0001); there was no effect of exposure plant (χ^2^_2_ = 1.9, *P* = 0.37). While it is not possible to calculate a traditional R^2^ value with a linear model of this nature, the adjusted pseudo-*R* ^2^ is 0.23, suggesting a rather poor association between oviposition and larval development. Moreover, it indicates that there are multiple other factors influencing a female *B. cockerelli*'s oviposition decisions.

## Discussion

Theoretically, herbivorous insects should prefer host plant species that will maximize their fitness, while eschewing those hosts that result in lower fitness. The mechanism for these choices is formally described by various hypotheses that link preference for oviposition to performance of the insect. Preference-performance type hypotheses predict maximum oviposition on plants with optimal larval success. However, occasionally an insect species will choose different plant species for oviposition from those that they can complete development on [64]–[67]. This can be the result of multiple factors including a mistake on the mother's part and a differential suitability among life stages. In the latter, a plant species that is suitable for feeding by adults may not provide optimal nutrition for nymphal development. Our results suggest that *Bactericera cockerelli* have host plant preferences that cannot be explained by performance alone.

In a previous study comparing *Bactericera cockerelli* from California and Texas on both bell pepper and tomato, Liu and Trumble [40] demonstrated that the psyllids from Texas performed better than the California population with respect to survivorship, growth index, and development time. Additionally, the California population showed more variability with respect to host plant use, with greater performance on tomato than on pepper, even though the psyllids had been collected on pepper. In this study, we expanded upon the Liu and Trumble results by rearing and testing *B. cockerelli* on different plant species including potato, bell pepper and tomato. This expanded study revealed some intriguing new patterns. First, three-choice bioassays indicated that psyllids have clear host plant preferences and these exist for both settling behaviors and oviposition. Interestingly, settling preference was a function of the host plant alone, while the natal plant influenced choice for oviposition. This pattern was further noticed in behavioral observations where a complicated but similar pattern of host and natal plant influence was observed. Overall, these patterns suggest that psyllids prefer their natal host plant, both for settling and oviposition.

Variability in *B. cockerelli* host preference has management implications. Prager et al. (in press) noted that spatial distribution in *B. cockerelli* varies among crops and subsequently developed individual sampling plans for each of tomato (Prager et. al, in press) and pepper [68], while Butler and Trumble [69] published a sampling plan for potatoes. These results help explain this pattern, which likely results from a combination of *B. cockerelli*'s preference for the particular plant and other factors such as the plant's age. The latter has been shown to influence choice among potato varieties (Prager et. al, in press). Additionally, this solidifies the concept that sampling and management strategies must be crop specific.

To determine if behavioral preferences reflect *B. cockerelli* performance (development), we conducted no-choice bioassays with *B. cockerelli* reared on one of three host plant species and exposed to a given host plant species. These experiments were conducted on *B. cockerelli* collected from two distinct geographic locations, and with genetically distinct haplotypes. Similar to the three-choice bioassays, we found that natal plant affects oviposition. These experiments further indicated that oviposition behavior is also a characteristic of haplotype. This would indicate that *B. cockerelli* from different locations differ in their host preferences. We also found that the proportion of eggs that hatched and the ability to develop from egg to adult are also influenced by haplotype and/or natal and exposure host plant species. Overall, our studies indicate a complicated relationship in which *B. cockerelli* exhibit a preference for their natal host plant, but also have preferences associated with haplotype.

Although the majority of herbivorous insect species are thought to be specialists [2], [19], [70], plasticity in host plant selection is not an unknown phenomenon. For example, cotton leaf worms (*Spodoptera littoralis*) strongly favor their larval host plant when it is presented as an option [71]. Similarly, many studies in Lepidoptera [72]–[75] reveal differential host plant use. We found that *B. cockerelli* can use potato, tomato and pepper; however, they do not use them equally or with the same success. Broadly, the finding of differential host plant use is similar to the findings of Wallis [76] who examined *B. cockerelli* from the Colorado-Nebraska-Wyoming area on multiple host plants and found fewer eggs on eggplants, potato and tomato relative to many weedy plant species. Wallis found that among the crop species examined, potato (*Solanum tuberosum* L.) had fewer eggs than both tomato (*Solanum esculentum* Mill) and bell pepper (*Capsicum frutescens* L.), which is similar to the patterns we found for California collected psyllids. *B. cockerelli* have also been examined in New Zealand, where Martin [77] reports on eight suitable host plants including bell pepper, tomato and potato. Martin cites bell pepper as the most suitable of all plants examined, equal to tomato and to potato. Interestingly, *B. cockerelli* in New Zealand are invasive and genetic studies suggest they are the same haplotype as those from California [78].

In keeping with the findings reported here, several other studies have found variation in *B. cockerelli* performance on different host plants. Yang and Liu [38] examined potato psyllids on multiple host plants and found that egg to adult survival differs between eggplant and bell pepper, with adults emerging approximately two days sooner when eggs were laid on eggplant. Yang and Liu also found that females start to oviposit approximately 8 to 9 days after emergence and that this preoviposition period was not influenced by host plant. Knowlton and Thomas [37] also examined multiple plant species and reported substantial differences in the ability of eggs to hatch or develop. In some instances, nymphs reached second or third instar before dying, findings that would result in non-zero GI values less than one, which are what we calculated for *B. cockerelli* on various host plants.

The response of an insect to a host plant is not necessarily restricted to oviposition or settling. An insect might demonstrate numerous behavioral responses to a host plant including feeding. We examined such responses via a series of observational bioassays and found that host plants influenced most behaviors. The exception was that no effect was found in the walking behavior and the probing behavior was only significant as duration. Perhaps the most important behavioral trend we detected was with respect to time spent off leaf, where *B. cockerelli* reared on pepper spent substantially more time off the leaf than those reared on tomato. This is in opposition to feeding-like behaviors (feeding and probing) which are more common when *B. cockerelli* were reared on tomato or when pepper was not involved with the assay (i.e. reared on tomato and tested on potato or tomato). While we did not detect a significant effect of resting when measured as duration, when examined as a frequency, there was a significant effect seemingly driven by *B. cockerelli* reared on pepper resting less frequently regardless of the exposure plant. Taken as a whole, the behavioral observations suggest that potato psyllids reared on pepper are less “settled” and may be more likely to search for alternate hosts. The results of this experiment would be well suited to follow up studies using the electrical penetration graph technique that can specifically measure and quantify feeding and probing events [57].

In our no-choice bioassays, we found only a weak association between development and oviposition. This would suggest that while *B. cockerelli* choose to oviposit on more suitable host plants, they are rather poor at making these decisions and are influenced by many other factors. Additionally, the finding that nymphs can develop on nearly all the plants offered, the exception being *B. cockerelli* from California reared on pepper and presented potato, would indicate that the cost of a “mistake” may be relatively low in this species. This may be particularly important if availability is variable and optimal plant species are not available, as might be the case with annual crop species or if *B. cockerelli* originally evolved to use uncultivated species of plants.

We found that natal plant influences future host plant choice decisions. However, this trend only exists when haplotype is also considered. In order to conduct these experiments, we reared *B. cockerelli* on multiple host plants, which necessitated switching them from the host they had been collected and previously reared on. To minimize the possibility of maternal affects, we waited a minimum of three generations before using insects from these new colonies. Interestingly, the haplotype/geographic origin differences persisted. That is, when *B. cockerelli* were reared on a host plant species and exposed to that same species, there was generally a preference for that species. This may indicate that *B. cockerelli* learn to prefer the plant they develop on. However, oviposition and development also differed between haplotypes, and this may indicate that some portion of host plant range is genetic in basis and fixed. Such a genetic component may be a precursor to, or early indication of, local adaptation [13], [79]. If this pattern of variability in haplotype preferences occurs in other insects, then it could explain at least some of the exceptional variability reported in the literature that has led to considerable and contentious debate over topics such as Hopkins' host selection principle. Additionally, these combined results suggest that host choice in these psyllids is a combination of genetic and learned effects.

In studies with the moth *Spedoptera littoralis*, Thoming et al. (2013) found a preference for the larval host plant when it was present, despite its ability to use other hosts. This pattern reached an extreme when the moth was offered clover, a pattern that has been explained by the abundance of clover in the agroecosystem [71]. Potential mechanisms for such preferences for larval host plant are varied and controversial. For example, it has been suggested that information may transfer from larvae to adults via neural tissue that is retained throughout metamorphosis [80]–[83]. Another explanation is that chemicals from the larval host that are associated with diet “prime” the emerging adult and establish a preference [84]. Finally, Thöming et al. [71] suggest these behaviors may result from learning as larva or early adults. Unfortunately, the specific mechanisms for host choice are difficult to distinguish in our experiments, but are clearly in need of further investigation.

*Bactericera cockerelli* is a vector for the fastidious alphaproteobacterium *Candidatus* Liberibacter solanacearum [45], [48], [85]. In addition to its psyllid vector, CLso is associated with many solanaceous host plants, including eggplant, bell pepper, tomato and potato. Because CLso is known to cause disease in at least three of these species, especially potato, associations between CLso and potato psyllids have been the subjects of limited study. Gao et al. [86] examined the ability of *B. cockerelli* reared for multiple generations on tomato, potato and eggplant to transmit CLso into healthy plants and concluded that the ability to transmit CLso is independent of population. However, they also observed that *B. cockerelli* reared on bell pepper and eggplant caused more severe disease symptoms in both leaves and tubers of potato, and speculated that this was an effect of populations reared on different host plants. That conclusion is consistent with other studies that reported differences in development with respect to population [40] and tomato cultivar [87]. Here we report that not only do potato psyllids demonstrate preferences among suitable host plants, but also that both haplotype and natal plant can influence these preferences. This result suggests that the variability in CLso infection observed by Gao et al. [86] may result from differential use of the plants. Supporting this concept, Underwood [88] found different responses between closely related plant cultivars because of the length of insect feeding, while Rashed et al. [89] have shown that the number of potato psyllids on a plant influences disease symptoms and acquisition.

In their review of heritable insect symbionts, Hansen and Moran [90] discussed the possibility that symbionts enable insect hosts to utilize phloem and xylem sap as food. They further note that while it is unknown if symbionts play a role in determination of host range, such an effect would depend on the symbionts ability to change plant nutrient profiles. In a study of potato psyllids and CLso effects on tomato immune response genes, Casteel et al. [91] determined that *B. cockerelli* alone result in suppression of jasmonic and salicylic acid signaling. When tomato plants were exposed to both CLso and *B. cockerelli* there again was a reduction of defensive host responses. If CLso does reduce plant defense against insects, it may influence host range, especially if this response is variable among plants. In our studies, we used exclusively *B. cockerelli* infected with CLso. There was, though, variability in CLso haplotype within the Texas derived colonies while all *B. cockerelli* from California demonstrated the same CLso haplotype (Prager, unpublished data). These factors have two consequences. First, we are unable to assess the influence of CLso on host plant choice. Second, we cannot fully determine if CLso haplotype is contributing to the geographic origin/psyllid haplotype effect we observed in no-choice bioassays. These elements will require further study.

### Conclusions

This study has revealed some interesting and important patterns about *Bactericera cockerelli* host plant use. Both no-choice and three-choice bioassays confirmed *B. cockerelli*'s ability to use multiple common crop plant species as hosts. However, these bioassays also demonstrated that multiple factors influence host plant suitability, including haplotype and natal host plant. Moreover, no-choice bioassays suggest that preferences are only weakly associated with larval performance. These results suggest that *B. cockerelli* host range may vary due to local adaptation, that they may be “family” specialists rather than species or genus level specialists, or that there is a large contribution of learning to their host plant choices. A final consideration is that this study was conducted using potato psyllids from only two of the four known *B. cockerelli* haplotypes and all *B. cockerelli* were infected with CLso. Future studies are necessary to determine how additional haplotypes will factor into host preferences and whether CLso infection influences potato psyllids host plant selection.

## Supporting Information

Supporting Information S1 **Experimental setup used for three-choice bioassays.**
(TIF)
