# Supplementary figures and images for: Factors Influencing Host Plant Choice and Larval Performance in Bactericera cockerelli
Source: PLoS One. 2014 Apr 7;9(4):e94047. doi: 10.1371/journal.pone.0094047 (PMC3977993; doi:10.1371/journal.pone.0094047)

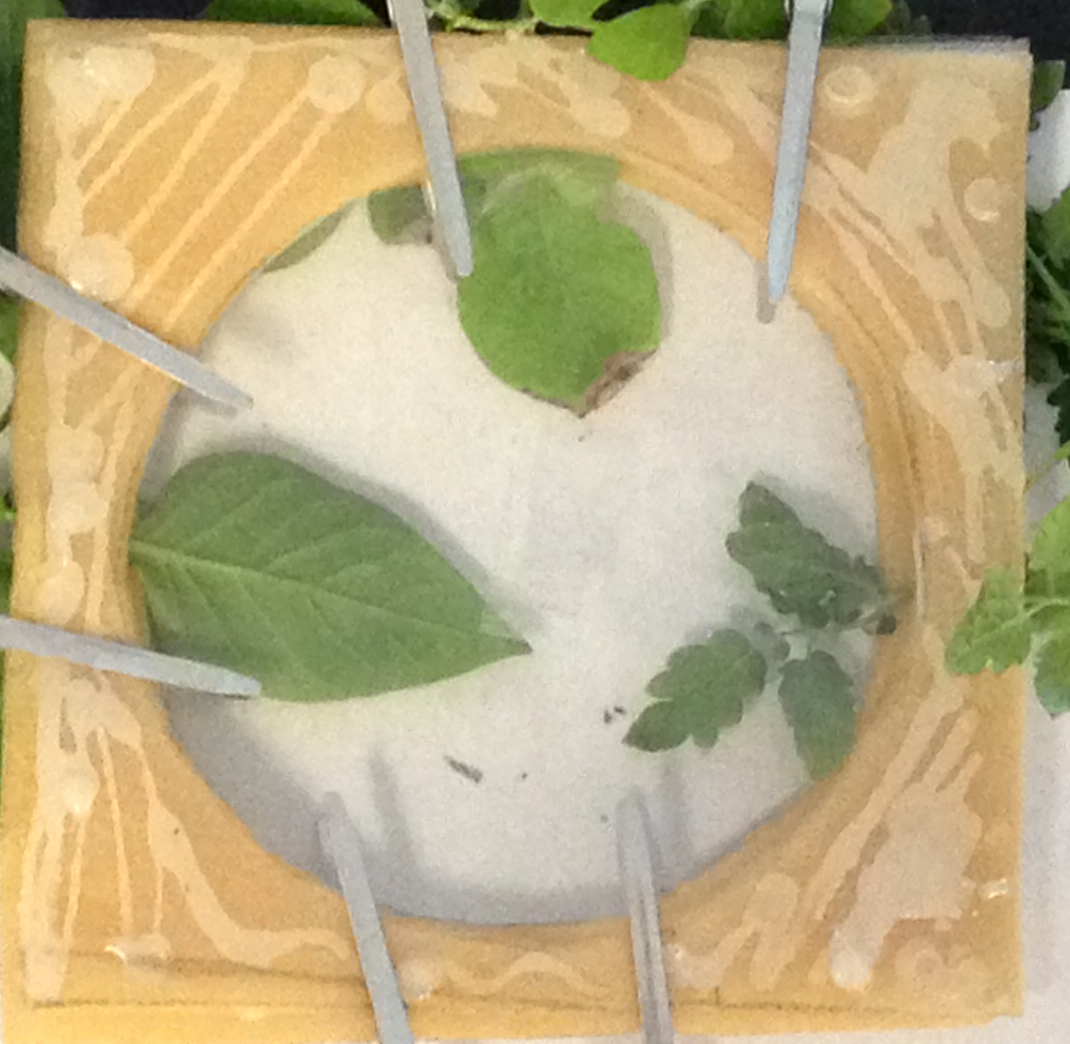

Supplement: Supporting Information S1 — Experimental setup used for three-choice bioassays. (TIF) [file pone.0094047.s001.tif]
